# COVID-19 und seine Umwelt: Von einer Geschichte der Humanmedizin zu einer ökologischen Medizingeschichte?

**DOI:** 10.1007/s00048-021-00299-3

**Published:** 2021-04-19

**Authors:** Leander Diener

**Affiliations:** Institut für Biomedizinische Ethik und Medizingeschichte, Universität Zürich, Winterthurerstr. 30, 8006 Zürich, Schweiz

**Keywords:** COVID-19, Ökologie, Anthropozän, Kreisläufe, Körpermodelle, COVID-19, Ecology, Anthropocene, Cycles, Body Models

## Abstract

Dieser Beitrag ist Teil des Forums COVID-19: Perspektiven in den Geistes- und Sozialwissenschaften. Medizingeschichte wird gegenwärtig mit wenigen Ausnahmen als Geschichte der Humanmedizin geschrieben. COVID-19 und andere zoonotische Infektionskrankheiten legen allerdings nahe, Medizingeschichte grundsätzlich ökologischer zu denken und nicht-menschliche Akteur*innen sowie verschiedene „Umwelten“ miteinzubeziehen. Der vorliegende Beitrag diskutiert mögliche Ansätze für eine ökologische Medizingeschichte, die der Überlagerung mehrerer Krisen gerecht wird und aktuelle Dringlichkeiten aufgreift.

## Abstract (en)

This paper is part of the Forum COVID-19: Perspectives in the Humanities and Social Sciences. The history of medicine is mostly written as a history of human medicine. COVID-19 and other zoonotic infectious diseases, however, demand a reconsideration of medical history in terms of ecology and the inclusion of non-human actors and diverse environments. This contribution discusses possible approaches for an ecological history of medicine which satisfies the needs of several current and overlapping crises.

Die Medizingeschichte basiert auf einer einfachen und meist unausgesprochenen Prämisse: Im Fokus des Interesses steht praktisch ausschließlich die Humanmedizin.^1^ Entsprechend sind die großen Themen der jüngeren Medizingeschichte – Sozialgeschichte der Medizin, Patient*innengeschichte, Körpergeschichte, Geschlechtergeschichte, Krankheitsgeschichte – in anthropomorphen Kontexten verortet. Nichtmenschliche Akteur*innen tauchen nur zuweilen als epistemische Objekte in einem bestimmten Experimentalsystem auf – beispielsweise im Fall von Louis Pasteur (Latour 1988) und Robert Koch (Gradmann 2005) oder in der Erforschung und Produktion von Wirkstoffen (Haller 2012). „Natürliche“ Umwelten werden entweder als externe Faktoren untersucht, zum Beispiel bezüglich spezifischer klimatischer Bedingungen (Sellers 2018) oder beim Studium von Überlebensstrategien prekärer Organismen als innere Milieus, die feindlichen externen Milieus gegenübergestellt werden (z. B. Geroulanos & Meyers 2018; Heggie 2019). Die leitenden Körpermodelle sind entsprechend maschinelle Körper (Sarasin 2001; Rabinbach 1990), bakteriologische Körper (Berger 2009a) oder homöostatische Körper (Kury 2012; Jackson 2013): Körper also, die auf einer bestimmten Konzeption der Abgeschlossenheit von Organismen basieren. Diese Körpermodelle lassen sich schließlich mit Gewinn auf bestimmte politische Agenden hin untersuchen (für den bakteriologischen Körper siehe z. B. Sarasin et al. 2007).

Als sich im Zuge eines weit um sich greifenden *Anthropological Turn* in den 1970er und 1980er Jahren aufregende neue Perspektiven auf geisteswissenschaftliche Themen entwickelten, wurde auch die Medizingeschichte davon erfasst (Güttler 2019: 236). So wurde beispielsweise der traditionelle Anthropozentrismus (oder vielleicht eher: Androzentrismus) der althergebrachten Institution „Medizin“ mit feministischen und postkolonialen Ansätzen hinterfragt, etwa mit dem Argument, dass die moderne Medizin auf einem bestimmten problematischen männlichen, westlichen und weißen Standardkörper beruhe. Die gleichzeitige Ökologisierung des Theorieninventars in historischen Disziplinen, das heißt das Aufkommen von situierenden und lokalisierenden Herangehensweisen, hätte durchaus den tradierten, nun kritischen Standardkörper um das Attribut „human“ erweitern können. Warum dies nicht geschehen ist, soll jedoch nicht Gegenstand dieser kurzen Erörterung sein. Jedenfalls zog die Medizingeschichte anders als die Medizinanthropologie und auch die Wissenschaftsgeschichte nicht so bereitwillig mit, als es um die Integration der Natur und der Umwelt in den Untersuchungsbereich und um die entsprechende ökologische Re-Konzeptualisierung des Körpers und des Menschlichen ging: Die medizinische Umwelt wurde primär sozial gedacht. Und während sich die Medizinanthropologie und die Wissenschaftsgeschichte bereits seit Jahren mit dem diagnostizierten Zeitalter des Anthropozäns und den damit verbundenen Folgen beschäftigt, arbeitet sich die Medizingeschichte weiterhin an dem sich aus der Körpergeschichte der 1980er Jahre ergebenden Spannungsfeld zwischen essenzialistischem Biologismus und Sozialkonstruktivismus ab.^2^

Auf den ersten Blick scheinen Medizingeschichte und die Geschichte des Anthropozäns wenig miteinander zu tun zu haben; geht es hier um anscheinend relativ geschlossene gesellschaftliche Aushandlungsprozesse von Gesundheit und Krankheit, wird dort unter anderem das menschliche Eingreifen in die Erdgeschichte thematisiert. Wie genau die Veränderung der Umwelt medizinhistorisch aufgearbeitet werden kann und soll, ist eine offene Frage. Eine Zeitgeschichte der Medizin – und darauf läuft die Frage nach dem Anthropozän früher oder später hinaus – beschäftigt sich jedenfalls momentan eher mit anderen Faszinationsthemen: den Neurowissenschaften, hochtechnisierter Spitzenmedizin, der epidemiologischen Transition oder mit AIDS und anderen Infektionskrankheiten in einem Schnittbereich zur Medizinanthropologie. Die AIDS-Pandemie ist hier ein besonders aufschlussreiches Beispiel, zumal der HIV-Erreger relativ früh mit einer Form von Mensch-Tier-Übertragung in Verbindung gebracht wurde (Grmek 1990: 141–155). Ebenfalls war relativ früh klar, dass im Zentrum der Geschichtsschreibung von AIDS vor allem die soziale Konstruktion der Krankheit stand (Fee & Fox 1992; McKay 2017). Diese historische Interpretation von AIDS war sowohl auf eine historiografische als auch eine soziopolitische Gewichtung der Zeit zurückzuführen und prägt die einschlägige Literatur bis in die Gegenwart.

Der Übergang ins 21. Jahrhundert brachte mit einer Häufung von epidemischen Infektionskranken wie SARS und Ebola schließlich aufs Tapet, wovor vonseiten der Tierseuchenüberwachung seit Jahrzehnten gewarnt worden war. Die maßlose Landnahme und damit einhergehende Verdrängung von Wildtieren sowie die Zunahme von Jagd und Konsumation provozierte eine Häufung sogenannter Zoonosen im ausgehenden 20. und frühen 21. Jahrhundert (Hahn et al. 2000: 613). Die COVID-19-Pandemie stellt lediglich das jüngste Beispiel dar, so kann man in verschiedenen Expertisen lesen; weitere zwischen Menschen und Tieren zirkulierende Viruserkrankungen werden mit großer Wahrscheinlichkeit folgen. Die drohende Gefahr von Zoonosen, aber auch die irreversible Realität von Antibiotikaresistenzen in der Landwirtschaft führte zum gesundheitspolitischen Projekt von One Health, in dem verschiedene internationale Organisationen dazu aufforderten, von der strikten Trennung in Human- und Veterinärmedizin abzusehen und der wechselseitigen Verflochtenheit der Gesundheit von Menschen und Tieren mehr Beachtung zu schenken. Das Wellcome-Trust-finanzierte historische Projekt zu One Health von Abigail Woods, Michael Bresalier, Angela Cassidy und Rachel Mason Dentinger untersuchte vor diesem Hintergrund historische Zusammenhänge, in denen Tier und Mensch enggeführt wurden (Woods et al. 2018). Abgesehen von diesem dezidiert auf die Schnittmenge von Human- und Veterinärmedizin fokussierenden Projekt widmeten sich weitere historische Projekte den Kreisläufen, die Mensch und Tier verbinden. Beispielhaft ist etwa Hannah Landeckers Arbeit über den Metabolismus von nicht-menschlichen und menschlichen Akteur*innen, in dem Abfallprodukte aus der industriellen tierischen Nahrungsmittelproduktion zirkulieren (Landecker 2019).

Dass die Jahre 2020 und 2021 im Zeichen der COVID-19-Pandemie standen und stehen, lässt die Ausrichtung einer Medizingeschichte hinterfragen, in der Tiere höchstens peripher interessieren. Welche Agenda wird verfolgt, wenn Tierkörper und damit auch die natürliche Umwelt größtenteils ausgeklammert werden – oder vielmehr, wie müsste eine Agenda aussehen, die das nicht tut? Und wichtiger: Kann man sich das überhaupt noch leisten? Mit dieser zweiten Frage hängt eine weitere größere Aufgabe zusammen, die sich die Medizingeschichte stellen müsste: Wie findet die Medizingeschichte Anschluss an einige der brennendsten Themen des 21. Jahrhunderts, zum Beispiel die grundlegende Umgestaltung der Umwelt und die Klimaerwärmung? Eine Geschichtsschreibung, die einer gewissen Orientierungsleistung in der Gegenwart verschrieben ist, leistet im besten Fall einen Beitrag zur Diskussion von aktuellen Fragen anhand historischer Zusammenhänge. COVID-19 als Zoonose gibt den Blick frei auf eine globalisierte Menschheit, die nicht nur sozial auf der Welt, sondern auch biologisch mit der Welt verknüpft ist. Diese Interdependenzen können Angst machen, aber auch Grund zur Hoffnung geben – Hoffnung darauf, dass die Position des Menschen in der Welt wieder verhandelbar wird, was beispielsweise angesichts der Machtstellung neoliberaler Institutionen und einer verfestigten Ressourcenlogik im 21. Jahrhundert kaum mehr für möglich gehalten wurde. Das ökonomische Wachstumsparadigma und die Idee des freien Marktes stellten im Sommer 2020 nicht nur die Rolle des Staates gegenüber einem überforderten Gesundheitswesen zur Debatte. Infrage gestellt wurde auch die Idee, dass sich menschliche Körper dank einem *pharmaceutical-technological fix* gegen alle möglichen Gefahren aus der Umwelt absichern können.

Diese Situation fordert gewissermaßen eine Neuverhandlung alter Gewissheiten in der Medizingeschichte. Wie könnte also eine ökologisch gedachte Medizingeschichte aussehen? Julie Livingston schlug ganz grundsätzlich vor, Medizingeschichte als „Vorgeschichte“ unserer neuen „Normalität der klimawandel-induzierten Ausnahmesituation“ neu zu denken (Langstaff 2020). COVID-19 erscheint gemäss Richard C. Keller lediglich als „Facette einer breiteren Erfahrung der spätkapitalistischen Moderne, oder des gegenwärtigen Anthropozän-Höhepunkts“ (Langstaff 2020). In diesem Sinne könnte zunächst empirisch nach den Auswirkungen historischer Formationen des Gesundheitswesens auf die Umwelt gesucht werden, etwa in Form von materiellen Kreisläufen (z. B. pharmazeutische Produkte wie Hormone im Grundwasser) oder von Abfallprodukten aus dem Gesundheitswesen (z. B. infektiöser Abfall). Dann könnte nach den gesellschaftlichen, kulturellen, politischen und institutionellen Bedingungen für diese Formationen gefragt werden. Anschließend müssten die Mechanismen und Infrastrukturen untersucht werden, die dafür verantwortlich waren, dass das Wissen über derartige Kreisläufe und ökologische Vernetzungen oft prekär war und zuweilen unterdrückt wurde. Solche Formen von Wissen beziehungsweise Nicht-Wissen lassen gesellschaftliche Reaktionen auf COVID-19, den Klimawandel und andere Problemlagen vergleichbar werden.

Laura J. Martin, Assistenzprofessorin für Environmental Studies, wies bereits im April 2020 darauf hin, dass man über die Ähnlichkeiten und Unterschiede zwischen der COVID-Pandemie und der Klimaerwärmung nachdenken müsste (Martin 2020). Wie kommt es, dass in einem Fall unzählige (tendenziell jüngere) Menschen gewaltige Veränderungen in ihrem Alltag in Kauf nehmen, während im anderen Fall grundlegende Änderungen in Produktion und Konsum nur zögerlich umgesetzt werden? Wie soll man sich zur Verschränkung der beiden Krisen verhalten, etwa hinsichtlich des Backlashs im Klimaschutz, der durch immense Infrastrukturprogramme und gelockerte Umweltschutzbestimmungen zur Stabilisierung der nationalen Wirtschaft in China und in den USA ausgelöst wird?

Eine weitere Möglichkeit könnte darin liegen, die der Medizin zugrunde liegenden Körpermodelle zu untersuchen – von menschlichen und nichtmenschlichen Körpern. Medizinische Körpermodelle und damit zusammenhängende Körperbilder sind ein zentraler Aspekt von Subjektivierung und politischer Agency. Auf diese Weise käme man schnell auf tierethische und tierrechtliche Diskussionen, aber auch auf soziale und politische Fragen: Körperbilder erlauben einen tiefgreifenden Blick auf das Selbstverständnis und die Handlungsfähigkeit von politischen Subjekten, wie etwa Philipp Sarasin anhand der „reizbaren Maschine“ zeigt.^3^ Je nach politischem Projekt muss danach gefragt werden, auf welche Weise Körperbilder Entscheidungen beeinflussen. In diesen Formen des Selbstbezugs treten immer bestimmte Verhältnisse zu „natürlichen“ und „kulturellen“ Umwelten zutage. Körpermodelle wie der maschinelle, der bakteriologische oder der homöostatische Körper operieren beispielsweise in ungesunden, feindlichen Umwelten. In diesem Überlebenskampf ist der Spielraum für Interventionen in die Umwelt entsprechend groß. Hier überlagern sich also anthropologische Selbstbezüglichkeiten, Umweltverhältnisse, Wissensordnungen und Handlungslogiken in einer spezifischen Art und Weise. Ausgehend von COVID-19 als Zoonose wäre es daher für unsere Gegenwart naheliegend, Medizin und vor allem die verhandelten Körpermodelle im Zeichen einer kritischen ökologischen Medizingeschichte zu überdenken und sich von der strikten Trennung in Organismen (menschliche Körper) und deren Umwelten (nicht-menschliche Körper und „natürliche“ Umwelten) zu lösen beziehungsweise deren intrikate Verstrickungen in den Fokus zu rücken.

## Footnotes

1 In einer COVID-19-Spezialausgabe der Zeitschrift *Centaurus* vom Mai 2020 behandeln nur zwei von insgesamt 15 Beiträgen die Rolle von Tier und Umwelt in der Pandemie (D’Abramo & Neumeyer 2020; Green 2020). Gleiches gilt mit zwei Ausnahmen für die Sektion „Second Look: Pandemics“ der Zeitschrift *Isis* (Second Look: Pandemics 2020). Im ersten „Forum COVID-19“ der Zeitschrift *NTM *behandelt keiner von sechs Beiträgen die Rolle von Tier und Umwelt in der Pandemie. Im Gegensatz dazu widmet sich eine Ausgabe der Zeitschrift *Environmental History* dezidiert dem Gebot der Stunde, das Menschliche neu zu denken (Alagona et al. 2020). Während für die *environmental history* seit einiger Zeit Grundlagentexte vorliegen (Chakrabarty 2009; Ingold 2011), fehlt ein Text mit vergleichbarer Dringlichkeit für die Medizingeschichte.

2 Diese Charakterisierung der Medizingeschichte ist als heuristische Polemik zu verstehen und wird natürlich keineswegs allen Ansätzen gerecht. Zwischenstufen in Richtung einer ökologischen Medizingeschichte bilden Projekte, die den abgeschlossenen Körper historisieren und problematisieren. Dazu gehören beispielsweise Projekte, die sich mit Wirt-Parasit-Interaktionen in der Medizin (z. B. Berger 2009b; Méthot & Dentinger 2016), mit Umweltkonzepten in den Lebenswissenschaften (z. B. Wessely & Huber 2017; Benson 2020), mit interspezifischen Wissenstransfers (z. B. Kohler 1994; Creager 2001; Roelcke 2009), mit Antibiotikaresistenzen (z. B. Condrau 2009; Podolsky 2015; Gradmann 2016; Keck 2018), mit der industriellen Lebensmittelproduktion oder verschiedenen Formen von Krankheitsökologie (z. B. Anderson 2004; Honigsbaum 2016; Seth 2018; Lynteris 2019; Engelmann & Lynteris 2020) auseinandersetzen. Ebenso aufschlussreich sind Arbeiten zur „Biomolekularisierung“ des Lebens (z. B. Kay 1993; Keller 1995; de Chadarevian 2002; Grote 2019; Morange 2020), die einen von mehreren wissenshistorischen Kontexten für den medizinhistorischen Anthropozentrismus bilden.

3 Mit der Agency von Tieren beschäftigt sich eingehend das interdisziplinäre Feld der Human-Animal Studies. Untersucht wird hier die Interaktion von Tieren und menschlichen Gesellschaften, häufig entweder als intime Beziehungen mit oder industrielle Nutzung von Tieren – beides Themen mit einer bestimmten politischen Agenda (Kompatscher 2019: 12–13). Aus der Perspektive nichtmenschlicher Akteure lassen sich demgemäß soziale Ordnungen menschlicher Gesellschaften beleuchten (Roscher 2012). Einflussreich waren hier insbesondere Donna Haraway und Bruno Latour, indem sie die kulturell geprägten Grenzziehungen zwischen Tier und Mensch kritisch auf epistemische Hegemonien befragten (Latour 1995; Haraway 2007; Eitler & Möhring 2008).
